# Changes in Microbial Communities, Including both Uncultured and Culturable Bacteria, with Mid-Ocean Ballast-Water Exchange during a Voyage from Japan to Australia

**DOI:** 10.1371/journal.pone.0096274

**Published:** 2014-05-09

**Authors:** Akiko Tomaru, Masanobu Kawachi, Mikihide Demura, Yasuwo Fukuyo

**Affiliations:** 1 Asian Natural Environmental Science Center, The University of Tokyo, Tokyo, Japan; 2 Environmental Biology Division, National Institute for Environmental Studies, Ibaraki, Japan; CSIR- National institute of oceanography, India

## Abstract

We assessed changes in the microbial communities in ballast water during a trans-Pacific voyage from Japan to Australia that included a mid-ocean ballast-water exchange. Uncultured (i.e., total) and culturable bacteria were counted and were characterized by using denaturing gradient gel electrophoresis (DGGE). There was a clear decrease over time in numbers of uncultured microorganisms, except for heterotrophic nanoflagellates, whereas the abundance of culturable bacteria initially decreased after the ballast-water exchange but then increased. The increase, however, was only up to 5.34% of the total number of uncultured bacteria. Cluster analysis showed that the DGGE profiles of uncultured bacteria clearly changed after the exchange. In contrast, there was no clear change in the DGGE profiles of culturable bacteria after the exchange. Multidimensional scaling analysis showed changes in microbial communities over the course of the voyage. Although indicator microbes as defined by the International Convention for the Control and Management of Ships' Ballast Water and Sediments were occasionally detected, no coliform bacteria were detected after the exchange.

## Introduction

The history of non-indigenous aquatic organisms being dispersed by cargo vessels goes back more than 100 years [1]. One of the more famous reports by Ostenfeld [2] is about a bloom of the diatom *Biddulphia* (now *Odontella*) in the North Sea in 1903–1907. Ostenfeld also cited the hypotheses of Fabre-Domergue [3] and Cotton [4] that exotic species of algae might be introduced by currents, directly by vessels, or with mixed cultivation of young oysters or lobsters.

The cargo ship *John Bowes* was the first to have built-in water ballast tanks installed, in 1852 [5]. Even in the 1800s, several investigators attempted to determine whether planktonic organisms would survive in ballast tanks during voyages (reviewed in [6]). Beginning in 1973, as a result of the International Conference on Marine Pollution, the International Maritime Consultative Organization began investigating the organisms transported by ballast water [7]. At that conference, ballast-water problems were considered and Resolution 18 was adopted. This resolution was based on a draft resolution calling for research into the effects of ballast-water discharge containing epidemic-causing disease bacteria; the draft had appeared in response to an outbreak of gastroenteritis in children in about 1972 (Y. Sasamura, personal communication). In the early 1970s, however, outbreaks of gastroenteritis were caused not by bacteria but by norovirus and rotavirus pathogens [8], [9]. Nevertheless, this was the impetus for surveys that focused on the relationship between bacteria and ballast water.

Although ballast water has been sampled and studied since vessels were first built with ballast-water tanks, probably the first samples known to have been collected from a ship's ballast tanks at the end of a voyage were those reported by Medcof (1975) [10]. A subsequent review by Rosenthal (1976) [11], as quoted by the International Maritime Organization (IMO) (1998) [12], pointed out that ballast water was only one of the candidate risk factors for the accidental introduction of exotic organisms; aquaculture operations were just as likely to spread exotic species.

The control of exotic marine species became a global movement in 1982 with the United Nations Convention on the Law of the Sea [13]. The IMO began a concrete and substantial effort to address the ballast-water problem presented by Canada in a paper at the 26th session of the Marine Environment Protection Committee (MEPC26) in September 1988 concerning the presence and implications of foreign organisms in ship ballast water discharged into the Great Lakes [12]. As if to reconfirm this mission, in Rio de Janeiro in 1992 the United Nations Conference on Environment and Development recognized the issue of the introduction of non-indigenous species via ballast water in ships (Agenda 21, Chapter17) [14]. At MEPC33 in October 1992, the committee looked at the extent to which the 1991 Guidelines for Preventing the Introduction of Unwanted Organisms and Pathogens from Ships' Ballast Waters and Sediment Discharges were being implemented [12].

It is believed that in the last 20–30 years many invasive species have been introduced via ballast water [6], [15]–[18]. Thus, the movement of ballast water and ballast tank sediments is currently regarded as one of the most important mechanisms for the transfer of aquatic non-indigenous species [18], [19]. Although the introduction of exotic organisms has a major economic impact on the aquatic environment, aquaculture, and other industries in some countries, it is not clear which invasive species are transported by which vectors. Nevertheless, because the IMO suspected and recognized ballast water in ships as a potential vector for invading species, in February 2004 it adopted the International Convention for the Control and Management of Ships' Ballast Water and Sediments (BWM) [20]. This convention includes technical standards and requirements for the control and management of ships' ballast water that are currently awaiting ratification. The IMO standard will be phased in over time, depending on the age and size of vessels. It is based on an allowable discharge of viable organisms within specified size categories [20].

During the time when BWM was under negotiation, ballast-water exchange was being used to reduce the risk of introducing alien invaders. Ballast-water exchange is the replacement of ballast water taken up in coastal areas with water from the open ocean. Oceanic organisms generally do not survive when released into the coastal or fresh waters of a destination port [21]. This method will continue to be used during the phase-in period until all vessels meet the IMO standard.

Ship-borne microbial transport could result in the introduction of toxic dinoflagellates, which was one of the concerns of the MEPC27 [12], [22], although it is not clear whether these species have been transported in the past. An epidemic caused by *Vibrio cholerae* O1 in Latin America in 1991 [23] accelerated concerns over the problem of ballast water and pathogenic microorganisms; cholera was then placed on the “Ten Most Unwanted” list of diseases in the Global Ballast Water Management Program (GloBallast) [24]. Even though the *V. cholerae* O1 responsible for the 1991 outbreak was thought to have come from ballast water, this could not be proven [25]. Officials of the Pan American Health Organization believed that the bacteria first arrived with a freighter, which apparently released contaminated bilge water into the harbor at Lima, Peru [26]. Bilge water is defined in “ANNEX I: Regulations for the prevention of pollution by oil” of the International Convention for the Prevention of Pollution from Ships, 1973 (MARPOL); “sewage” is defined in “ANNEX IV: Regulations for the prevention of pollution by sewage from ships” [27]. Although there is no chance today of bilge water having pathogens, there was no retroactive application of Annex IV at the time of its adoption. It is therefore possible that bilge water was mixed with sewage at the time of discharge into the harbor at Lima, Peru. Ultimately, there no clear cause was identified for the *Vibrio cholerae* O1 outbreak in Latin America.

With this as background, the Annex to BWM, “Regulation D-2, Ballast Water Performance Standard,” regulates not only plankton but also microbes. The indicator microbes are toxicogenic *Vibrio cholerae* (O1 and O139), *Escherichia coli* and intestinal *Enterococci*. These bacteria are basically detected by live counting methods using plate counts [20]. Because bacterial control was an addendum to BWM, the IMO delayed its adoption of BWM [28], [29].

In assessing microbes in ballast water, most studies have focused solely on pathogens (e.g., [19], [30]–[34]). Tomaru et al. (2010) [35] studied the effect of mid-ocean ballast-water exchange on bacterial abundance and community structure in ballast water, but their research was limited to uncultured bacteria. In this study, we investigated the qualitative and quantitative changes in members of the microbial loop community resulting from a mid-ocean ballast-water exchange during a trans-Pacific voyage from Japan to Australia. We observed both uncultured and culturable bacteria, including those regulated by BWM.

## Materials and Methods

### Ethics statement

No specific permits were required for the field studies. The ship's owners, the company operating the ship, the customers transporting cargo on the ship, and Fukushima Prefecture's local fishermen's union reviewed and approved our experimental protocol before we initiated sampling. No permit was necessary for sampling on the high seas because of “freedom of the seas,” a principle in the United Nations Convention on the Law of the Sea (1982). The field studies did not involve endangered or protected species. In addition, our terms of agreement with the ship's owners, the company operating the vessel, and the customers transporting cargo on the vessel prohibited us from publishing any details or identifiable cargo information, such as the names of ports or the name of the vessel. All of the information about this voyage that we are permitted to present is in Table 1.

**Table 1:** Sampling schedule and sample identifiers.

Date	Day of voyage	Latitude	Longitude	SW series	BW series	
				(Surface only)	Surface	Bottom
**18 June**	**0**	**37°53′N**	**141°01′E**	**SW01**		
**20 June**	**1**	**37°53′N**	**141°01′E**	**SW02**	**BW01S**	**BW01B**
**21 June**	**2**	**34°54′N**	**142°40E**		**BW02S**	**BW02B**
**22 June**	**3**	**29°18′N**	**142°40E**		**BW03S**	**BW03B**
**23 June**	**4**	**23°42′N**	**145°56E**		**BW04S**	**BW04B**
24 June*	**5**	**17°44′N**	**147°53E**	**SW03**	**BW05S**	**BW05B**
**25 June**	**6**	**12°49′N**	**149°27E**		**BW06S**	**BW06B**
**26 June**	**7**	**07°15′N**	**150°60E**		**BW07S**	**BW07B**
**27 June**	**8**	**01°09′N**	**152°31E**		**BW08S**	**BW08B**
**28 June**	**9**	**04°28′S**	**153°21E**		**BW09S**	**BW09B**
**29 June**	**10**	**10°24′S**	**154°25E**		**BW10S**	**BW10B**

Seawater (SW) series and ballast-water (BW) series. Asterisk (*) indicates mid-ocean ballast-water exchange on day 5.

### Sample collection

This study was conducted aboard a coal-carrier (58,098 t gross) on a voyage from Japan to Australia (Table 1). Ballast water was taken on at a port in Japan and then discharged before the reloading of ballast water in mid-ocean (17°42′5″N–17°23′8″N) on day 5 of the voyage. The water in each ballast tank was exchanged. Ballast-tank water samples (“BW” series) were collected with a vacuum pump; samples of ballast-water from the surface (BWS) and the bottom (BWB) were collected from a manhole and an on-deck sounding pipe by using hoses or pipes, respectively (Figure S1). Seawater samples (“SW” series) were taken with a bucket over the side of the vessel. The ballast-water sampling schedule is shown in Table 1. Day 5 samples were collected after the ballast-water exchange. The main tank sampled was no. 7 TST P (capacity, 760 m^3^) (Figure S2). Samples were numbered in sequence.

For bacterial analyses, about 15 L of seawater or ballast water was collected into disposable sterilized plastic bags (a single sample). For plankton analysis (data not included in this study), about 300 L of water was filtered through 40-µm and 100-µm-mesh-size plankton nets, or about 20 L was collected directly into buckets. Although ballast water samples were shared by several researchers, there were only two or three researchers aboard, because merchant vessels generally have no space for research personnel. Our ballast-water sampling systems were washed with clean fresh water after each ballast-water sampling.

### Temperature and salinity

Water samples for temperature and salinity measurements were collected into buckets from the vacuum pump. Temperature and salinity were immediately measured with a portable conductivity/pH meter (WM-22EP; DKK-TOA Co., Osaka, Japan).

### Direct counts of microbes

For direct counts of microbes or uncultured bacteria, seawater subsamples were fixed with glutaraldehyde (final concentration, 1% by volume) within 1 h of sampling. For counting virus-like particles (VLPs), 0.1–1 mL of fixed sample was filtered onto a 0.02-µm-pore-size Anodisc filter (Whatman International Ltd., Maidstone, UK) and stained with SYBR Gold (Invitrogen Corporation, Carlsbad, CA, USA) [36]. For total bacterial counts (TBCs), 2 mL of fixed sample was stained with 4′,6-diamidino-2-phenylindole dihydrochloride n-hydrate (DAPI; Wako Pure Chemical Industries, Ltd., Osaka, Japan) and passed through a 0.2-µm-pore-size black Nuclepore filter (Whatman International Ltd., Kent, UK) [37]. Heterotrophic nanoflagellates (HNFs) and autotrophic nanoflagellates (ANFs) were counted by using a double-staining epifluorescence technique. For this technique, 5–100 mL of fixed sample was stained with DAPI and fluorescein-4-isothiocyanate (FITC; Dojindo Laboratories, Kumamoto, Japan) and then filtered through a 0.8-µm-pore-size black Nuclepore filter (Whatman International Ltd., Kent, UK) [38], [39]. All filters were placed on glass slides and stored at −20°C until the microbes were counted. VLPs and bacterial cells on filters were counted at 1000× magnification under an epifluorescence microscope (BX60; Olympus, Tokyo, Japan). HNFs and ANFs on filters were counted at 400× magnification under an epifluorescence microscope (BX60; Olympus). Counts were to at least 400 cells or over 20 fields of view.

### Counts of live microbes

Here, we define culturable bacteria are those bacteria detectable by plate count methods, because BWM specifies that plate count methods be used for detection and control of indicator microbes [20]. For counts of culturable bacteria, we followed the procedure proposed by the Marine Environment Protection Committee of Japan [40], [41], briefly summarized here. Plate counts of heterotrophic bacteria (HPCs) were performed by pipetting 100 µL of seawater sample or seawater sample diluted with sterilized phosphate buffer solution [42] containing NaCl (final concentration 0.3%) onto a Petri dish containing solid media, spreading the liquid sample by using sterilized spreaders, and allowing the plate to dry before inverting and incubating at room temperature (around 25°C). Colonies were counted 5 days later. Two media were used: Marine Agar 2216 (BD Difco, Detroit, MI, USA) for plate counts on seawater-based medium (HPC_sw_), and R2A Agar (BD Difco) for plate counts on freshwater-based medium (HPC_fw_). Because freshwater bacteria are able to survive in seawater, our aim was to investigate the fate of the bacteria culturable on freshwater-based medium after the ballast-water exchange.

Our counting protocol was based on the methods for counting coliforms (including *Escherichia coli*), enterococci, and *V. cholerae* O1 and O139, defined as indicator bacteria by BWM. However, we could not count enterococci onboard during the voyage because the solid medium used for their culture (m-Enterococcus Agar; Difco) does not keep for long periods of time. To measure the abundance of other indicator bacteria as defined by BWM, we did plate counts of coliforms, *E. coli*, and *V. cholerae* O1 and O139.

For counts of BWM indicator bacteria, water samples (1–100 mL) were passed through sterilized 0.2-µm-pore-size membrane filters (Millipore Corp., Bedford, MA USA). Each filter was placed on a Petri dish containing solid medium. In some cases, samples were not filtered but instead 100 µL of sample was spread on a Petri dish containing solid medium. Colonies of coliforms and *E. coli* were counted on XM-G Agar (Nissui Pharmaceutical Co. Ltd, Tokyo, Japan) after 24 h of incubation at 35±0.5°C. Possible*Vibrio cholerae* O1 and O139 colonies were counted on TCBS Agar (Nissui Pharmaceutical Co. Ltd) after 24 h of incubation at 35±0.5°C. These strains form small, yellow colonies on this medium as a result of sucrose fermentation; they are collectively referred to here as “the cholera group (CG)”. Five replicates at each dilution were spread on each type of agar plate. Bacterial colonies were counted onboard during the voyage.

Marine *Vibrio* species were identified in our laboratory onshore at the University of Tokyo. The yellow colonies were isolated and inoculated into 1% peptone broth (pH 8.6; BD Difco) at 35±0.5°C for 8 h to inhibit the growth of marine bacteria until the samples could be analyzed. Potential colonies of *V. cholerae* O1 and O139 were isolated and suspended in a saline solution. The cells in suspension were identified by reaction with O1 and O139 antisera by using the kits *Vibrio cholerae* AD “SEIKEN” and Cholerae immunoserum “SEIKEN”, respectively (Denka Seiken Co., Ltd., Tokyo, Japan).

### Denaturing gradient gel electrophoresis

Denaturing gradient gel electrophoresis (DGGE) was conducted by using the methods of Tomaru et al. (2010) [35]. The samples of uncultured bacteria used for DGGE were the same as those in the TBC sample series. Water samples of 0.5–5 L were passed through 47-mm-diameter, 10.0-µm-pore-size Nuclepore filters (Whatman International Ltd) within 2 h of sample collection. The filtrate was then passed through 47-mm-diameter, 0.2-µm-pore-size Nuclepore filters. The filters were frozen at −20°C until DNA extraction.

The culturable bacteria used for DGGE were colonies from Marine Agar 2216, the same as the HPC_sw_ series (see “Counts of live microbes” in the Results section). After the determination of HPC_sw_, these colonies were frozen at −20°C until DNA extraction.

For DNA extraction, the 0.2-µm filters or frozen colonies were sonicated in 0.2% sodium dodecyl sulfate – TE buffer (10 mM Tris [pH 8.0], 1 mM EDTA) for 5 min. The filter was then removed and DNA was extracted from the solution by using a Fast DNA Kit (Qbiogene, Inc., Carlsbad, CA, USA).

Bacterial 16S rDNA was amplified by PCR using primers GC341-F [43] and 907-R [44], which are intended to be specific for bacteria. The cycling conditions for PCR analysis were as follows: an initial denaturing step at 94°C for 1 min; 5 cycles of 94°C for 1 min, 63°C for 1 min, and 72°C for 1 min; 5 cycles of 94°C for 1 min, 58°C for 1 min, and 72°C for 1 min; 20 cycles of 94°C for 1 min, 55°C for 1 min, and 72°C for 2 min; and a final extension at 72°C for 7 min.

PCR products were purified with a QIAquick PCR Purification Kit (Qiagen, Valencia, CA, USA) and then loaded onto 6% polyacrylamide gels (acrylamide:N,N′-methylenebisacrylamide, 37∶1) with a denaturant gradient of 35% to 65% (where 100% is defined as 7 M urea and 40% [vol/vol] formamide). Electrophoresis was performed with the D Gene System (Bio-Rad, Hercules, CA, USA) using 1× tris-acetate-EDTA (TAE) running buffer (Bio-Rad) at 60°C for 13 h at 70 V. Gels were stained for 30 min in SYBR Green nucleic acid gel stain (1∶10,000 dilution; Molecular Probes, Eugene, OR, USA) and photographed with UV transillumination. DGGE banding patterns were converted to a binary matrix, and a Euclidean distance matrix was calculated from the binary data.

### Statistical analysis

The variations in bacterial communities over time leading up to, resulting from, and following the mid-ocean ballast-water exchange were assessed by multidimensional scaling (MDS) for composition changes and by unweighted pair-wise group arithmetic averages (UPGMA) for grouping. All calculations were performed with the R 2.3.1 statistical software package (http://www.r-project.org/). DGGE banding-pattern analyses were performed according to the methods of van Hannen et al. (1999) [45].

The effects of the mid-ocean water exchange on the bacterial community were analyzed by Wilcoxon test using the R 2.3.1 statistical software package (http://www.r-project.org/). Before the analysis, all data were square-root transformed [46].

## Results

### Water temperature and salinity profiles

**Figure 1:**
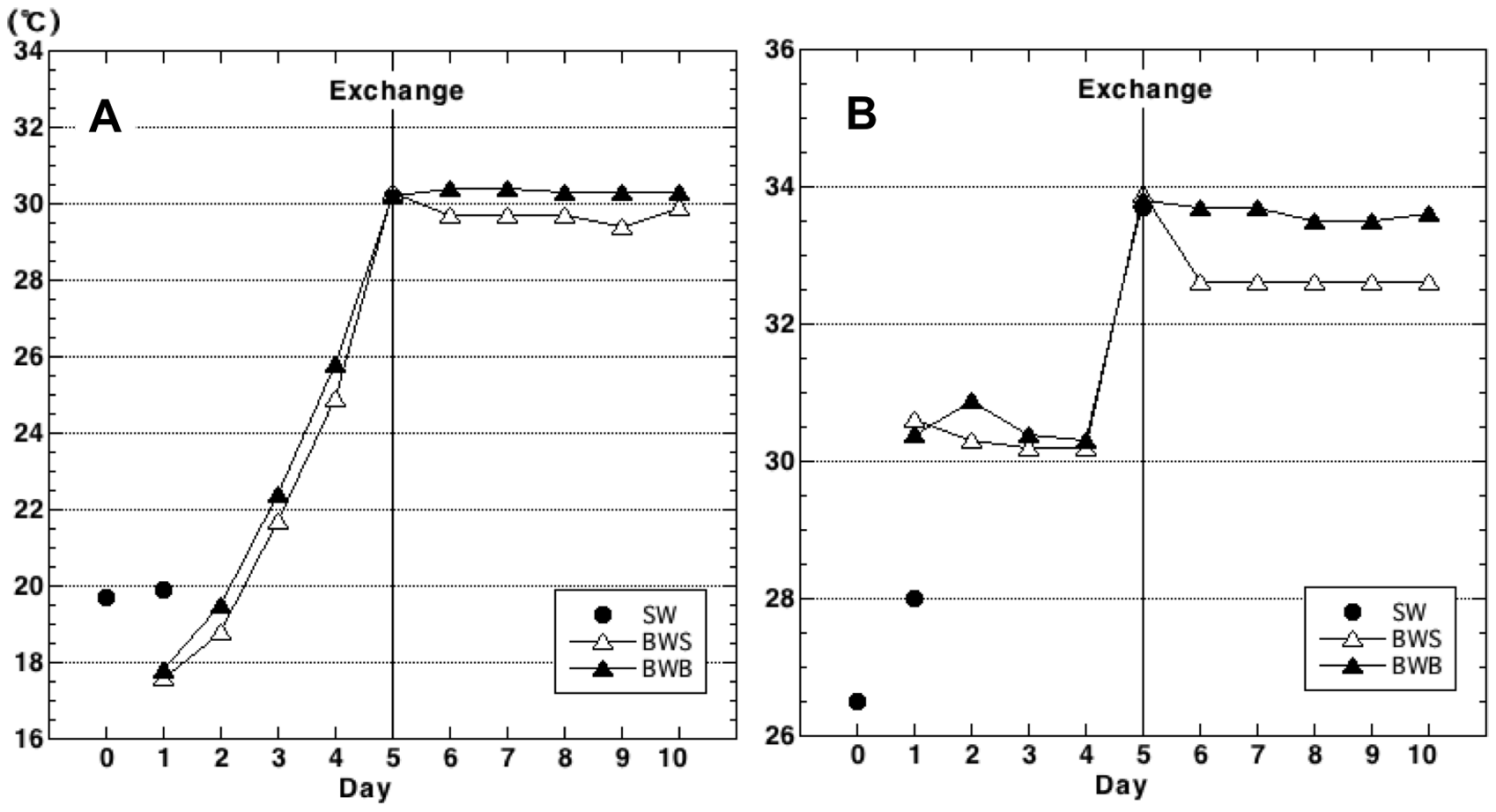
Temperature (A) and salinity (B) of surface seawater and ship's ballast water during a cruise from Japan to Australia in June 2005, roughly along longitudes 140°E to 155°E. Surface seawater (SW), closed circles; surface ballast water (BWS), open triangles; bottom ballast water (BWB), closed triangles. Mid-ocean ballast water exchange occurred on day 5 (Exchange).

The temperature and salinity of both surface and bottom ballast water showed similar trends over the course of the voyage (Fig. 1). Ballast-water temperature increased until the ballast-water exchange near the Equator and then remained constant at around 30°C. Salinity of both surface and bottom ballast water stayed at about 30.5 before the exchange and suddenly increased just after the exchange (day 5). The salinity remained at about 33.6 in the bottom water from day 6 onward, whereas it suddenly dropped in the surface water on day 6 and then remained constant at about 32.5 for the rest of the voyage.

The temperature of ambient seawater on day 1 was higher than those of day-1 ballast-water samples BW01S and BW01B (Fig. 1A), because sample SW02 (day 1) was collected from the surface of the ocean in the daytime. The salinity of SW02 was lower than those of BW01S and BW01B (Fig. 1B), possibly reflecting the effect of a river at the coast.

### Direct counts of microbes

**Figure 2:**
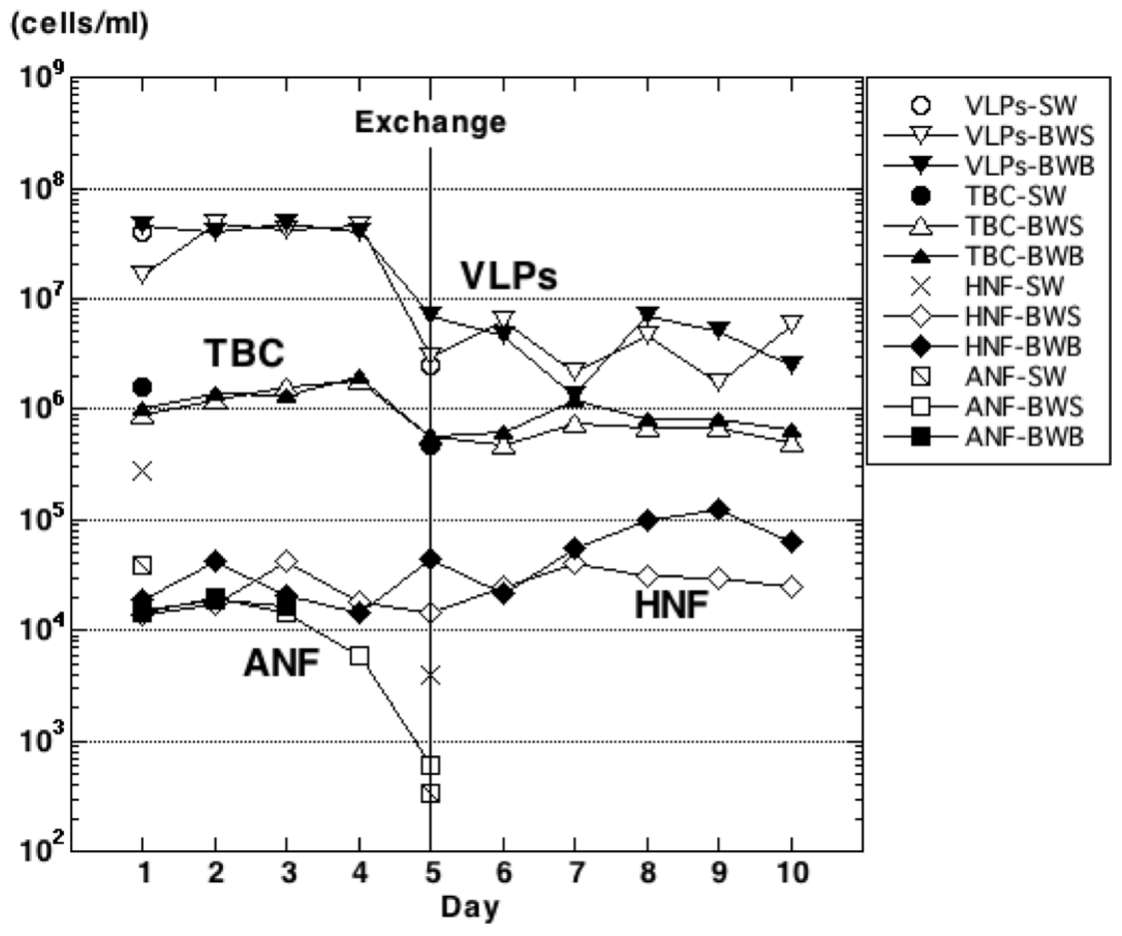
Microbial abundance as determined by direct counts, including virus-like particles (VLPs), total bacterial counts (TBCs), heterotrophic nanoflagellates (HNFs), and autotrophic nanoflagellates (ANFs). Microbes were counted in samples of seawater (SW) and in ballast-water surface (BWS) and bottom (BWB) samples. The mid-ocean ballast-water exchange occurred on day 5 (Exchange). Day 5 water samples were collected after the water exchange. No ANFs were detected in bottom ballast water after day 4 or in surface ballast water after day 6.

We used direct methods for TBCs and to count VLPs, HNFs, and ANFs. The results of direct counts showed similar trends for both surface and bottom ballast water throughout the voyage (Fig. 2).

The trends of VLP and TBC abundances were similar: constant before the ballast-water exchange and suddenly decreasing just after the exchange (samples BW05S and BW05B). The lower abundances of these groups remained constant after the exchange. Before the mid-ocean exchange of ballast water, TBCs were steady at approximately 1 × 10^6^ cells mL^−1^. After the exchange on day 5 of the voyage, TBCs rapidly decreased by one order of magnitude to approximately 1×10^5^ cells mL^−1^ (samples SW03, BW05S, and BW05B) and were fairly constant from day 8 onward. The difference between ballast-water TBCs before and after the exchange was significant (Wilcoxon test: surface, W = 24, *P*<0.01; bottom, W = 23, *P*<0.05). Before the mid-ocean water exchange, densities of VLPs were steady at approximately 1×10^7^ cells mL^−1^. After the exchange, VLP densities also suddenly decreased one order of magnitude to approximately 1×10^6^ cells mL^−1^ (samples SW03, BW05S, and BW05B) and were almost constant from day 8 onward. The decrease in VLPs after the exchange was significant (Wilcoxon test: surface, W = 24, *P*<0.01; bottom, W = 24, *P* = 0.01).

There was a significant increase in HNF densities after the ballast-water exchange (Wilcoxon test: surface, W = 1, *P*<0.05; bottom, W = 1, *P*<0.05). Even though the densities remained around the same order of magnitude, the abundances in both surface and bottom samples gradually increased after the exchange.

Densities of ANFs in surface ballast-water samples gradually decreased leading up to the ballast-water exchange and then further decreased immediately after the exchange (BW05S). ANFs were not detected in day 6 (BW06S) and later surface samples. In bottom samples, ANFs could be detected only in the first three samples (BW01B, BW02B, and BW03B) before the exchange.

### Counts of live microbes

**Figure 3:**
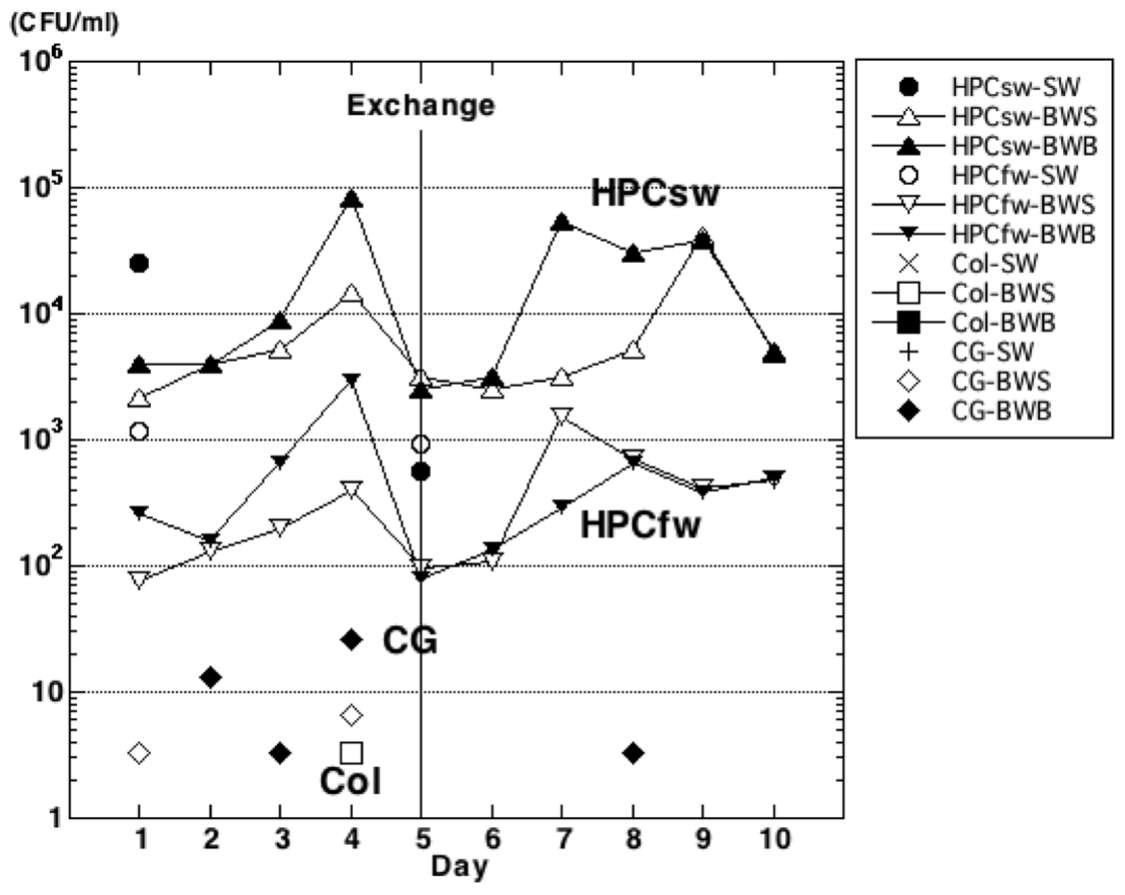
Densities of culturable bacteria in ballast water and natural seawater samples. Bacterial heterotrophic plate counts are classified as from seawater-based medium (HPC_sw_), freshwater-based medium (HPC_fw_), coliforms (Col), or members of the cholera group (CG). Microbes were counted in samples of ambient seawater (SW) and in ballast-water surface (BWS) and bottom (BWB) samples. Mid-ocean ballast-water exchange occurred on day 5 (Exchange). Missing symbols indicate days when no members of the coliform or cholera groups were detected. CFU, colony-forming unit.

We used plate-counting methods to monitor numbers of live bacteria. Plate counts were obtained for heterotrophic bacteria that were culturable on seawater-based medium (HPC_sw_) and freshwater-based medium (HPC_fw_). BWM indicator microbes were grouped as the cholera group or coliforms. On this voyage, we did not detect *V. cholerae* O1 or O139 or *E. coli* in any samples. The exchange of ballast water temporally affected HPC_sw_ and HPC_fw_: the abundance of both groups dropped just after the exchange in both surface and bottom ballast-water samples, and then the numbers increased (Fig. 3). There were no significant differences between densities before and after the exchange (Wilcoxon test. HPC_sw_: surface, W = 12, *P* = 1.00; bottom, W = 13, *P* = 0.914; HPC_fw_: surface, W = 6, *P* = 0.257; bottom, W = 15, *P* = 0.610). We calculated the ratios of plate (live) counts to direct (total) counts (HPC_sw_:TBC or HPC_fw_:TBC; Table 2). The ratio was higher in the HPC_sw_ series (0.24% to 9.84%) than in the HPC_fw_ series (0.01% to 0.20%).

**Table 2:** Numbers of heterotrophic bacteria as determined by the heterotrophic plate count (HPC) method, expressed as percentages of the direct total bacterial count (TBC).

Day of voyage	HPCsw/TBC (%)			HPCfw/TBC (%)		
	SW series	BW series		SW series	BW series	
		Surface	Bottom		Surface	Bottom
**1**	**1.57**	**0.24**	**0.40**	**0.07**	**0.01**	**0.03**
**2**		**0.33**	**0.28**		**0.01**	**0.01**
**3**		**0.33**	**0.66**		**0.01**	**0.05**
**4**		**0.81**	**4.16**		**0.02**	**0.15**
5*	**1.19**	**0.57**	**0.45**	**0.02**	**0.02**	**0.01**
**6**		**0.52**	**0.50**		**0.02**	**0.02**
**7**		**0.43**	**4.45**		**0.20**	**0.02**
**8**		**0.78**	**3.87**		**0.11**	**0.08**
**9**		**6.13**	**4.89**		**0.06**	**0.05**
**10**		**9.84**	**7.65**		**0.10**	**0.08**

SW, seawater samples; BW, ballast-water samples. Asterisk (*) indicates mid-ocean ballast-water exchange on day 5. Heterotrophic bacterial counts are from both freshwater-based medium (HPC_fw_) and seawater-based medium (HPC_sw_).

BWM indicator microbes were rarely found before the ballast-water exchange. Coliforms were detected in only one sample (BW04S), and the cholera group was detected sporadically in several surface and bottom samples (BW01S, BW04S, BW02B, BW03B, BW04B) (Fig. 3). After the ballast-water exchange only the cholera group was detected, and in only one sample (BW08B).

### DGGE profiles

**Figure 4:**
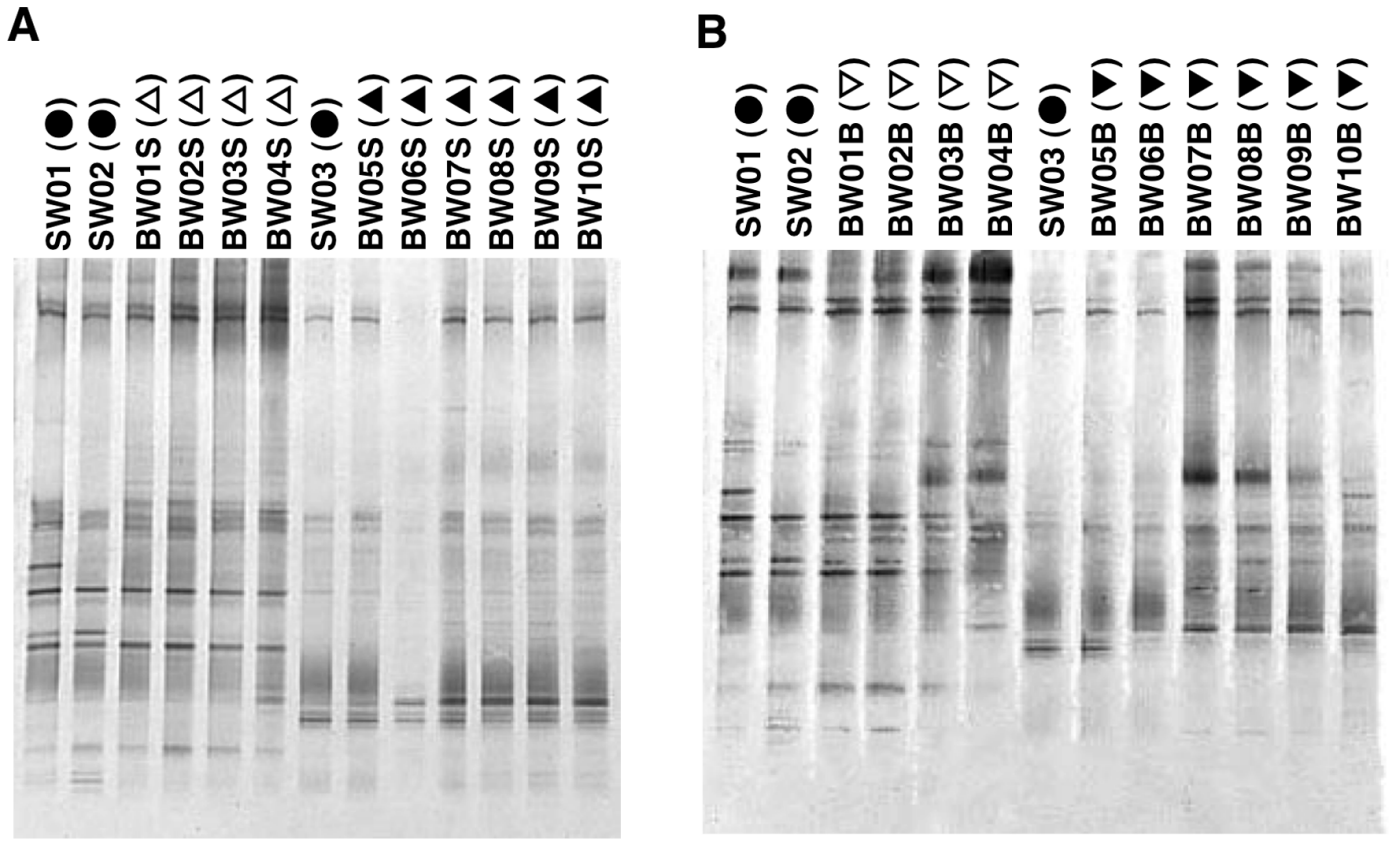
DGGE profiles of uncultured bacteria: DGGE band patterns. Refer to Table 1 for sample details. Surface (A) and bottom (B) samples, with surface seawater samples (SW01, SW02, SW03) included for comparison. Surface samples (A): seawater series (SW), closed circles; ballast-water (BW) series before mid-ocean exchange, open triangles; BW series after mid-ocean exchange, closed triangles. Bottom samples (B): seawater series (SW; surface only), closed circles; BW series before mid-ocean exchange, open inverted triangles; BW series after mid-ocean exchange, closed inverted triangles.

**Figure 5:**
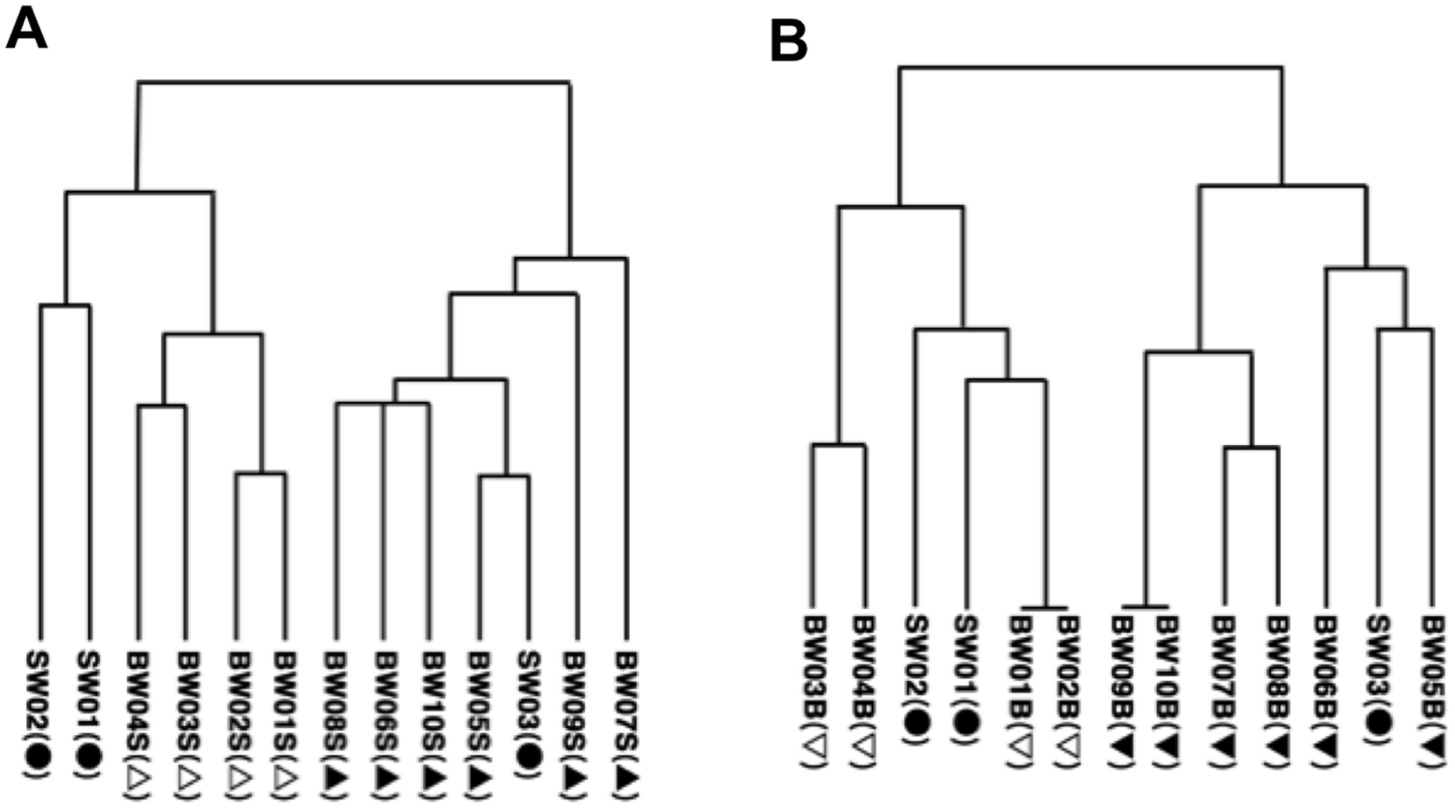
DGGE profiles of uncultured bacteria: unweighted pair-wise group arithmetic averages (UPGMA). Refer to Table 1 for sample details. Symbols as defined in Figure 4.

DGGE band patterns of uncultured bacteria in ballast-water samples are shown in Figure 4-A and B. The samples of uncultured bacteria used for DGGE had the same community composition as the samples for TBCs. The UPGMA results clearly show two clusters for both the surface (Fig. 5-A) and the bottom (Fig. 5-B) water samples: one from before the water exchange and the other after the water exchange.

**Figure 6:**
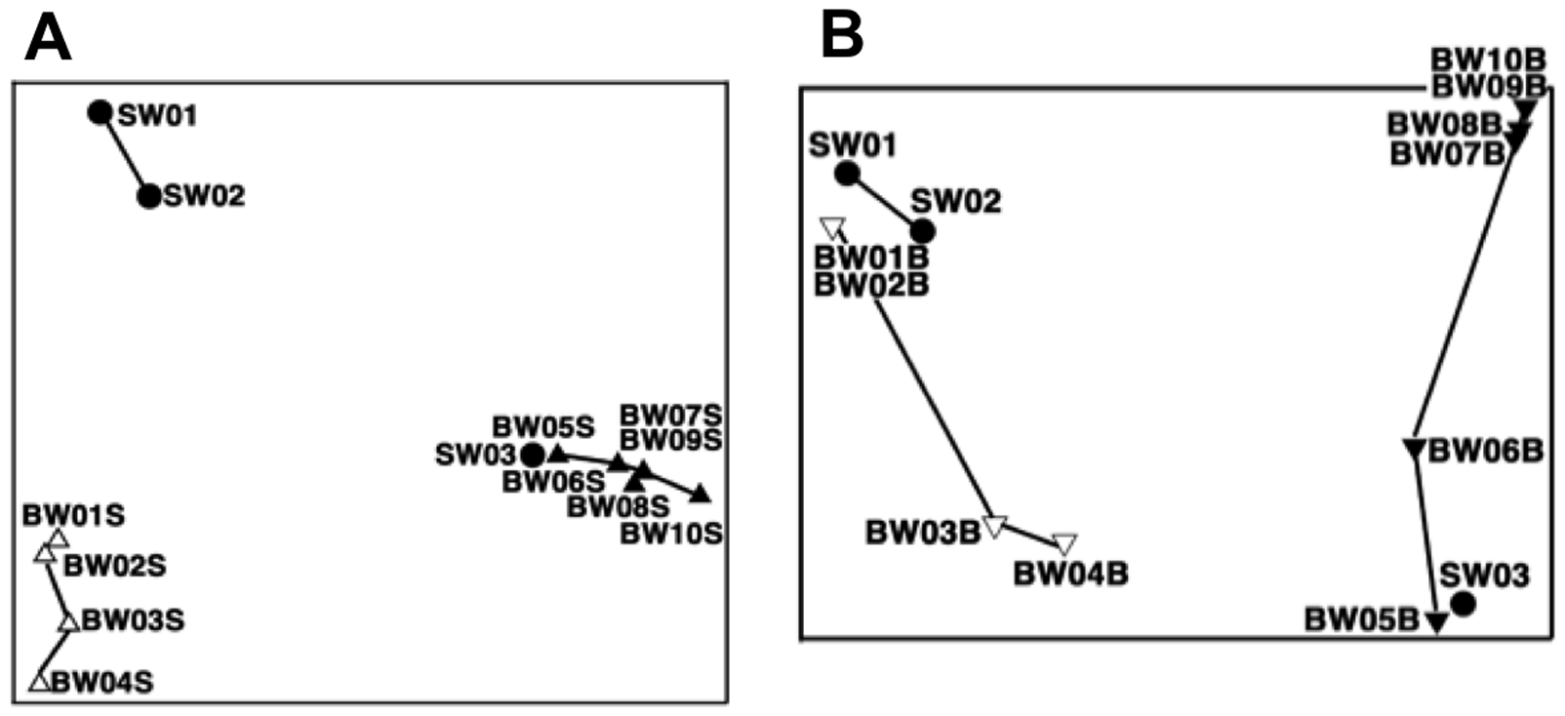
DGGE profiles of uncultured bacteria: multidimensional scaling analysis (MDS). Refer to Table 1 for sample details. Symbols as defined in Figure 4.

MDS analysis of DGGE band patterns of uncultured bacteria in surface-water samples (Fig. 6-A) revealed three groups: seawater from the port of departure (SW01 and SW02), samples taken before the exchange (BW01S to BW04S), and samples taken after the exchange (BW05S to BW10S), including mid-ocean water (SW03). The same analysis of the bottom water samples (Fig. 6-B) showed two groups: the samples taken before the water exchange (BW01B to BW04B) were grouped close to the port seawater samples (SW01 and SW02), and the samples taken after the exchange (BW05B to BW10B) were grouped with the mid-ocean water sample (SW03). The bacterial communities of both surface and bottom water changed slightly over time within each of these groups.

**Figure 7:**
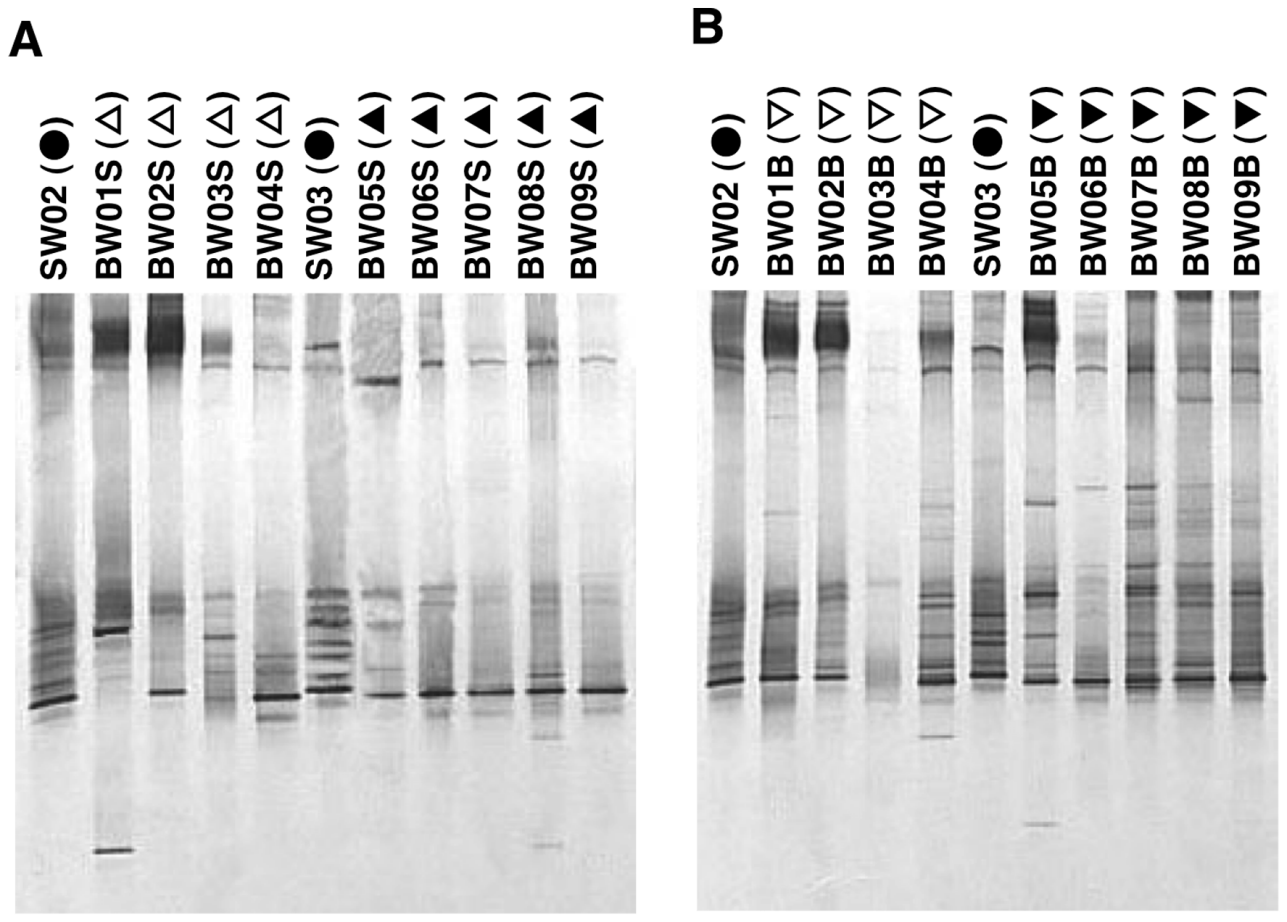
DGGE profiles of culturable bacteria: DGGE band patterns. Refer to Table 1 for sample details. Symbols as defined in Figure 4.

**Figure 8:**
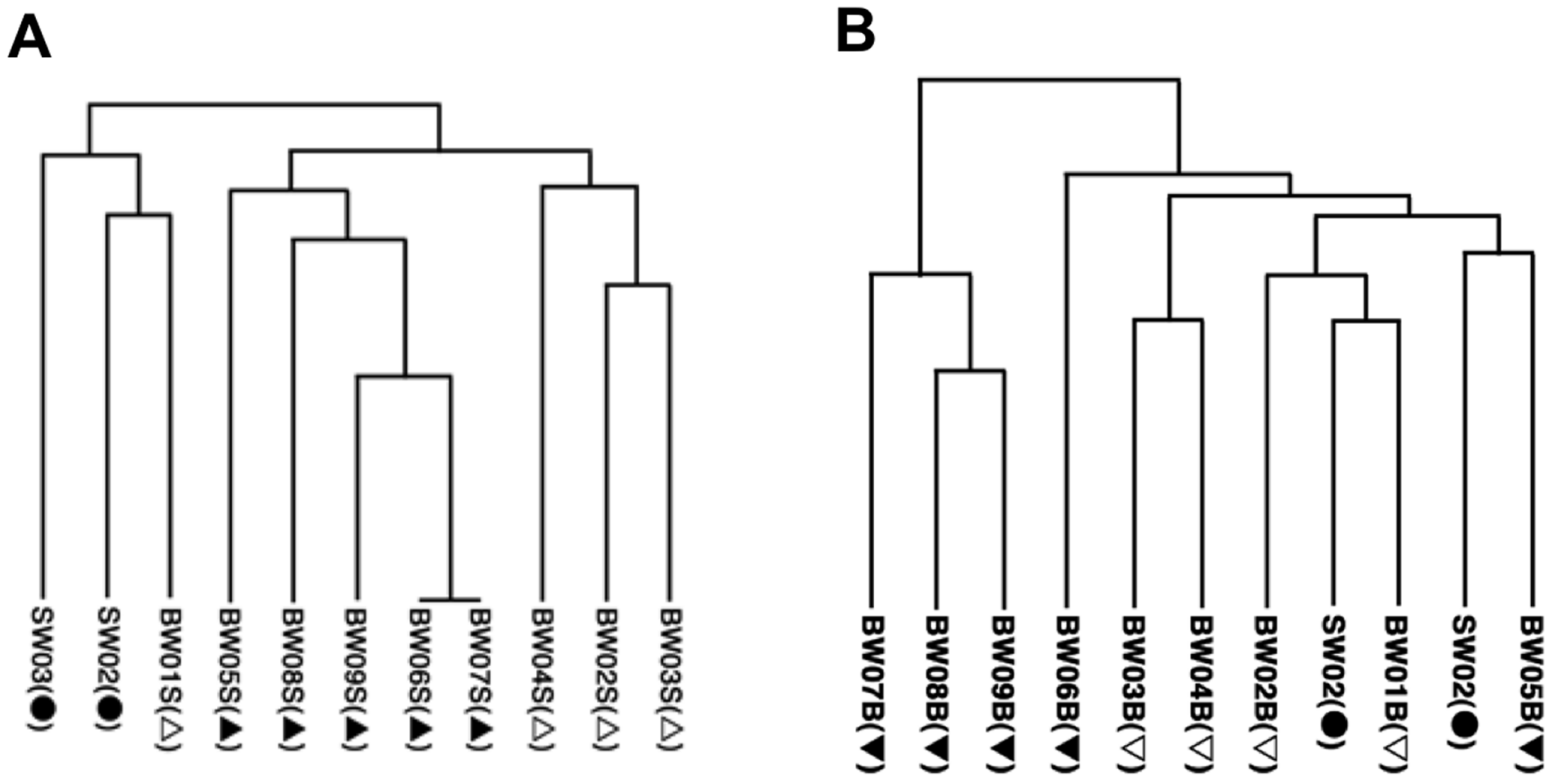
DGGE profiles of culturable bacteria: unweighted pair-wise group arithmetic averages (UPGMA). Refer to Table 1 for sample details. Symbols as defined in Figure 4.

The DGGE band patterns of culturable bacteria (Fig. 7-A and B) were clearly different from those of uncultured bacteria. These bacteria were from the colonies of culturable bacteria after the determination of HPC_sw_. The results of UPGMA suggest, but do not clearly show, two separate clusters (i.e. before and after the ballast-water exchange) for both the surface (Fig. 8-A) and the bottom (Fig. 8-B) samples. At the highest level, surface samples were separated into the home-port seawater samples (SW02 and SW03) along with BW01S, and the rest of the samples. At the next level they were roughly separated into samples from before and after the exchange, and these branches were nested. The grouping of the bottom samples was more complicated than that of the surface samples, and there was no clear trend relative to ballast-water exchange.

**Figure 9:**
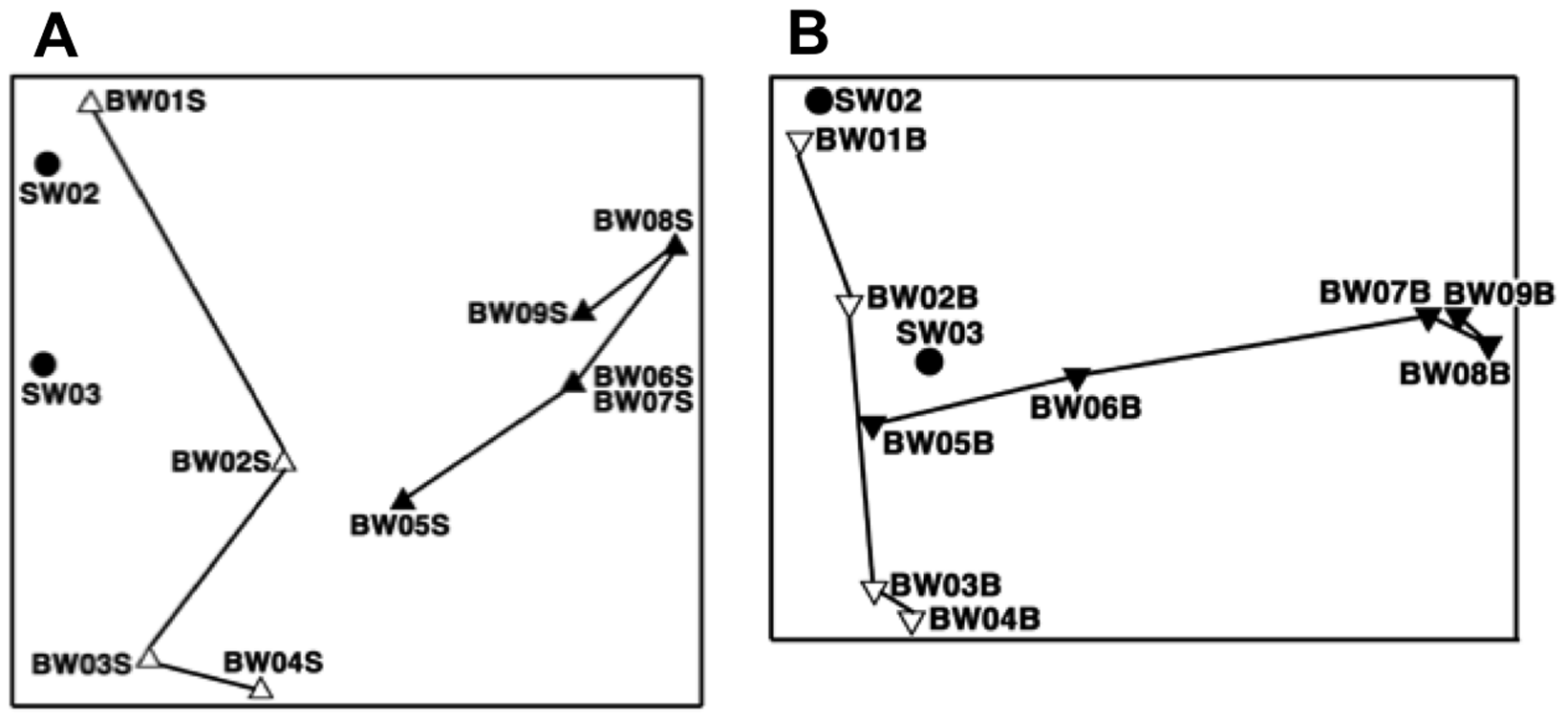
DGGE profiles of culturable bacteria: multidimensional scaling analysis (MDS). Refer to Table 1 for sample details. Symbols as defined in Figure 4.

The results of MDS analysis of culturable bacteria from both surface (Fig. 9-A) and bottom (Fig. 9-B) samples did not show any clear effect of the ballast-water exchange. However, the shifting sample positions in this analysis reflect changes over time in each bacterial community.

## Discussion

During the voyage, coastal ballast water was taken on before the start of the voyage and exchanged offshore well into the trip. The TBCs were about 1×10^6^ cells mL^−1^ before the ballast-water exchange and decreased to about 1×10^5^ cells mL^−1^ after the exchange (Fig. 2). There are extensive published data based on the direct counting method (e.g. [47]), and direct counts of heterotrophic bacteria are generally around 5×10^6^ cells mL^−1^ in estuaries, 1 to 5×10^6^ cells mL^−1^ in coastal waters, 5×10^4^ to 1×10^6^ cells mL^−1^ in offshore waters, and 1×10^4^ cells mL^−1^ in deep ocean waters [48], [49]. Our estimates of TBCs in ballast water and seawater are consistent with these values. The patterns of change in TBCs were similar in surface and bottom samples, and there were significant decreases in both types of sample after the ballast-water exchange. These trends are similar to those we observed in a series of samples collected during a winter voyage over the same track [35].

Drake et al. (2002) [50] reported no significant decrease in bacterial abundance after a ballast-water exchange in the North Atlantic Ocean. However, the abundances both before and after the exchange in their study were lower than in our present study (around 1×10^5^ cells mL^−1^), and thus no statistically significant differences were detected. If the concentration of organic matter was low in ballast-tank seawater in their study, the microbes were kept in a state of starvation. Bacteria in this state are known to decrease in size [51], and these smaller bacteria may not have been detected by the flow cytometer used in their study. Other surveys of ballast water had results similar to ours, with densities around 1×10^5^ to 1×10^6^ cells mL^−1^, which are reasonable numbers for marine environments [48], [49] and ballast water (e.g. [19], [35], [52]).

Generally, the concentration of VLPs in seawater ranges between 1×10^3^ and 1×10^9^ mL^−1^ [53]. Fuhrman (1999) [54] reported that the abundance of VLPs is typically 1×10^10^ L^−1^ in surface waters, or about 5 to 25 times the bacterial abundance. As expected, we observed similar dynamics for VLPs and TBCs, as did Drake et al. (2002) [50].

The changes that we observed in ANFs during this voyage are reasonable (Fig. 2). Because this voyage began after the ballast tanks were filled with coastal water, the abundance of ANFs in the tanks decreased over time in the absence of light, until the exchange of ballast water. In short, autotrophic species started dying earlier. Although mixotrophic species survived in the dark of the ballast-water tanks, they lost photosynthetic pigments and the abundance of ANFs decreased dramatically after day 3.

ANFs were next detected just after the water exchange; however, this time they disappeared more quickly than at the start of the voyage. One reason could be the low concentration of chlorophyll in mid-ocean water [53]; lower initial numbers after the water exchange would have resulted in ANFs reaching undetectable levels sooner by our counting method. Previous studies of ballast water report both increases and decreases in the abundance of phytoplankton (e.g. [55]–[58]). We interpret this to mean that the survival of ballast-water populations depends on the source of the water and the duration of the voyage.

Our results for HNFs show a significant difference between samples taken before and after the water exchange. However, the abundance in surface ballast water increased by a factor of 1.21 and in the bottom by 2.86 (Fig. 2). Some protists can survive for several weeks in ballast tanks (e.g. [59], [60]), and some, including algae, can transform to resting stages in ballast tanks (reviewed in [61]). Many species of protists form cysts (e.g. [62]), and this life-cycle stage generally plays an important role in either protection or dispersal of the species.

It is also possible that some protists, including HNFs, can survive and grow in ballast water because they are heterotrophic and detritus feeders. The ballast-water incubation hypothesis suggests that, in the darkness of ballast tanks, photosynthesis ceases, phytoplankton die, and zooplankton in turn starve and die; subsequently, this detritus in the system fuels bacterial production (e.g., [50]). We suggest that this detritus also fuels heterotrophic protists.

Some researchers contend that free-living microorganisms have no biogeography and are global in their distributions (e.g., [63], [64]); they therefore cannot be considered to “invade” environments. This idea has been explored with respect to ship-borne microorganisms [65]. We will not reiterate that discussion here, but in short, it contends that resolution of the microbial-ubiquity hypothesis is highly relevant to considerations of ballast management. Although the role of protists in ecosystems may not differ substantially among species, we must accept the possibility that protists in cyst form “hitchhike” in sea-going vessels and settle and grow in other areas as introduced species.

Our plate count abundance values for both HPC_sw_ and HPC_fw_ were higher than in previous reports (Table 2; reviewed in [66]). The increases in HPC_sw_ and HPC_fw_ before the ballast-water exchange closely paralleled the increasing water temperature. Although we did not measure dissolved or particulate organic matter concentrations, the concentration of dissolved organic matter is generally lower in open-ocean water than in coastal waters [67]; therefore, we speculate that water temperature controlled the increases in bacterial abundance. Numbers of both groups dropped in both surface and bottom ballast water just after the ballast-water exchange; both groups then increased, although not at the same rate (Fig. 3). Although the HPC_sw_ increased by about one order of magnitude after the ballast tanks were first filled (Fig. 3), this increase accounted for up to only 5.34% of TBC by day 9 of the voyage (Table 3). In short, the organisms detected by HPC_sw_ contributed a very small proportion of the TBCs. A study by Mimura et al. (2005) [33] using plate-counting methods could not confirm the effectiveness of ballast-water exchange in reducing the number of culturable bacteria, except on one of six voyages. Although their water samples were kept at 4°C until arrival in Japan, and the number and composition of bacteria in the samples at the time of analysis might have differed from those just after sample collection [68], our results were similar to theirs.

**Table 3:** Daily increase in numbers of heterotrophic culturable bacteria in ballast water as a percentage of direct total bacterial counts (TBC).

Day of voyage	Daily increase (%) relative to total bacterial counts in ballast water			
	HPCsw		HPCfw	
	Surface	Bottom	Surface	Bottom
**2**	**0.15**	**0.00**	**0.00**	**−0.01**
**3**	**0.08**	**0.37**	**0.00**	**0.04**
**4**	**0.53**	**3.71**	**0.01**	**0.11**
**5**	**−2.12**	**−14.29**	**−0.05**	**−0.51**
**6**	**−0.14**	**0.11**	**0.00**	**0.01**
**7**	**0.09**	**4.19**	**0.19**	**0.01**
**8**	**0.31**	**−2.91**	**−0.12**	**0.05**
**9**	**5.34**	**1.00**	**−0.05**	**−0.04**
**10**	**1.57**	**1.68**	**0.02**	**0.02**

Heterotrophic bacteria were counted by using the plate-count (HPC) method. Asterisk (*) indicates the day of the mid-ocean ballast-water exchange (day 5). Heterotrophic bacteria were counted on both freshwater-based medium (HPC_fw_) and seawater-based medium (HPC_sw_).

One of our goals was to detect indicator microbes by focusing on three pathogen groups targeted by the IMO for control. We could not detect enterococci, because the culture medium for this group of bacteria could not be preserved for inoculation during our voyage. Attention was focused instead on the other two BWM control pathogens: the cholera group and the coliforms—the group of enteric bacteria (including *E. coli*) that inhabit the intestinal tracts of humans and other warm-blooded animals [69]. The presence of these bacteria in water samples indicates fecal pollution. Taking this into consideration, our failure to detect coliforms in samples taken after the ballast-water exchange (Fig. 3) is reasonable.

Members of the genus *Vibrio* are highly abundant in aquatic environments, including in estuaries, marine coastal waters, and sediments, and in aquaculture settings worldwide [70]. We sporadically detected members of the cholera group in ballast-water samples (Fig. 3). This is reasonable, because the cholera group, including members of the genus *Vibrio*, contains sucrose-fermenting Gram-negative bacteria that are not likely to be difficult to culture from seawater. We could not, however, detect *V. cholerae* O1 or O139 at our laboratory after this voyage in any of the cholera-group samples cultured on TCBS agar. Other studies have obtained similar results [32], [33], [52], although Ruiz et al. (2000b) [34] reported detecting *V. cholerae* in plankton samples from all ships examined. We believe that the detection of *V. cholerae*, including serotypes O1 and O139, depends on the ballast water history.

The DGGE profiles of uncultured bacteria showed clear differences between samples taken before and after the ballast-water exchange (Figs. 4, 5 and 6), confirming that the ballast water had been replaced. DGGE profiles of culturable bacteria showed no clear difference between samples taken before and after the ballast-water exchange (Figs. 7, 8 and 9). The culture method used for detection seems to selectively detect cosmopolitan species and reveal artificial microbial communities. We suggest that the culture detection method is of limited value for assessing the effects of ballast-water exchange on the structure of heterotrophic bacterial communities.

Although the IMO adopted BWM in 2004, this convention has not yet come into effect. One of the reasons why it is still awaiting ratification is the absence of a proven ballast water management system [71], because just before the adoption of BWM it was decided to include bacterial controls [72], [73]. The indicator microbes identified by BWM are mainly pathogenic bacteria. Because individual countries control ship-borne pathogenic bacteria by using quarantine regulations, the pathogenic bacteria control provisions of BWM should be revised. It seems that both previous reports (e.g., [19], [30]–[34]) and our results show a very low probability of ship-borne pathogen distribution. Because it is difficult to revise the convention before its entry into force, additional ratifications by the interested administrations would be desired to amend the clause for bacteria criteria appropriately.

## Supporting Information

Figure S1 **Ballast water sampling.** Sampling at no. 7 TST P (capacity, 760 m3) ballast water tank (A); sampling surface ballast water (B); and bottom ballast water (C). In (B), 300 L of surface ballast water is being filtered through a plankton net for plankton analysis (data not included in this study). Water samples for bacteria were not filtered; instead 15 L of unfiltered ballast water was collected into a sterilized plastic bag. Ballast water samples were shared by several researchers, although only two or three scientists were aboard because merchant vessels have little extra room for survey personnel. Our sampling should not be against their voyage, so we could not have the control ballast tank (no ballast water exchange tank).
(TIF)

Figure S2 **Schematic of ballast water tank sampling.** Top side tank (TST), Water ballast tank (WBT), Port (P).
(TIF)
