# Supplementary figures and images for: Changes in Microbial Communities, Including both Uncultured and Culturable Bacteria, with Mid-Ocean Ballast-Water Exchange during a Voyage from Japan to Australia
Source: PLoS One. 2014 May 9;9(5):e96274. doi: 10.1371/journal.pone.0096274 (PMC4015909; doi:10.1371/journal.pone.0096274)

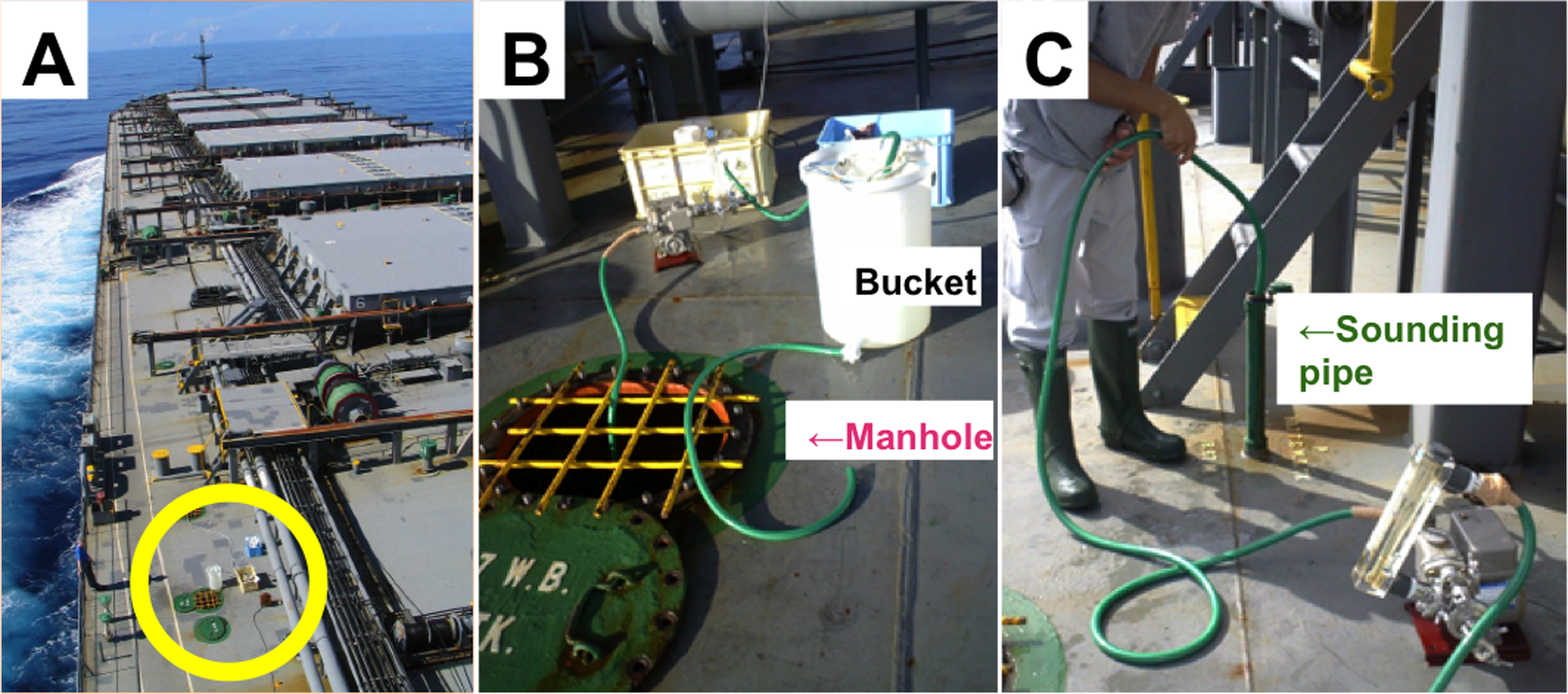

Supplement: Figure S1 — Ballast water sampling. Sampling at no. 7 TST P (capacity, 760 m3) ballast water tank (A); sampling surface ballast water (B); and bottom ballast water (C). In (B), 300 L of surface ballast water is being filtered through a plankton net for plankton analysis (data not included in this study). Water samples for bacteria were not filtered; instead 15 L of unfiltered ballast water was collected into a sterilized plastic bag. Ballast water samples were shared by several researchers, although only two or three scientists were aboard because merchant vessels have little extra room for survey personnel. Our sampling should not be against their voyage, so we could not have the control ballast tank (no ballast water exchange tank). (TIF) [file pone.0096274.s001.tif]

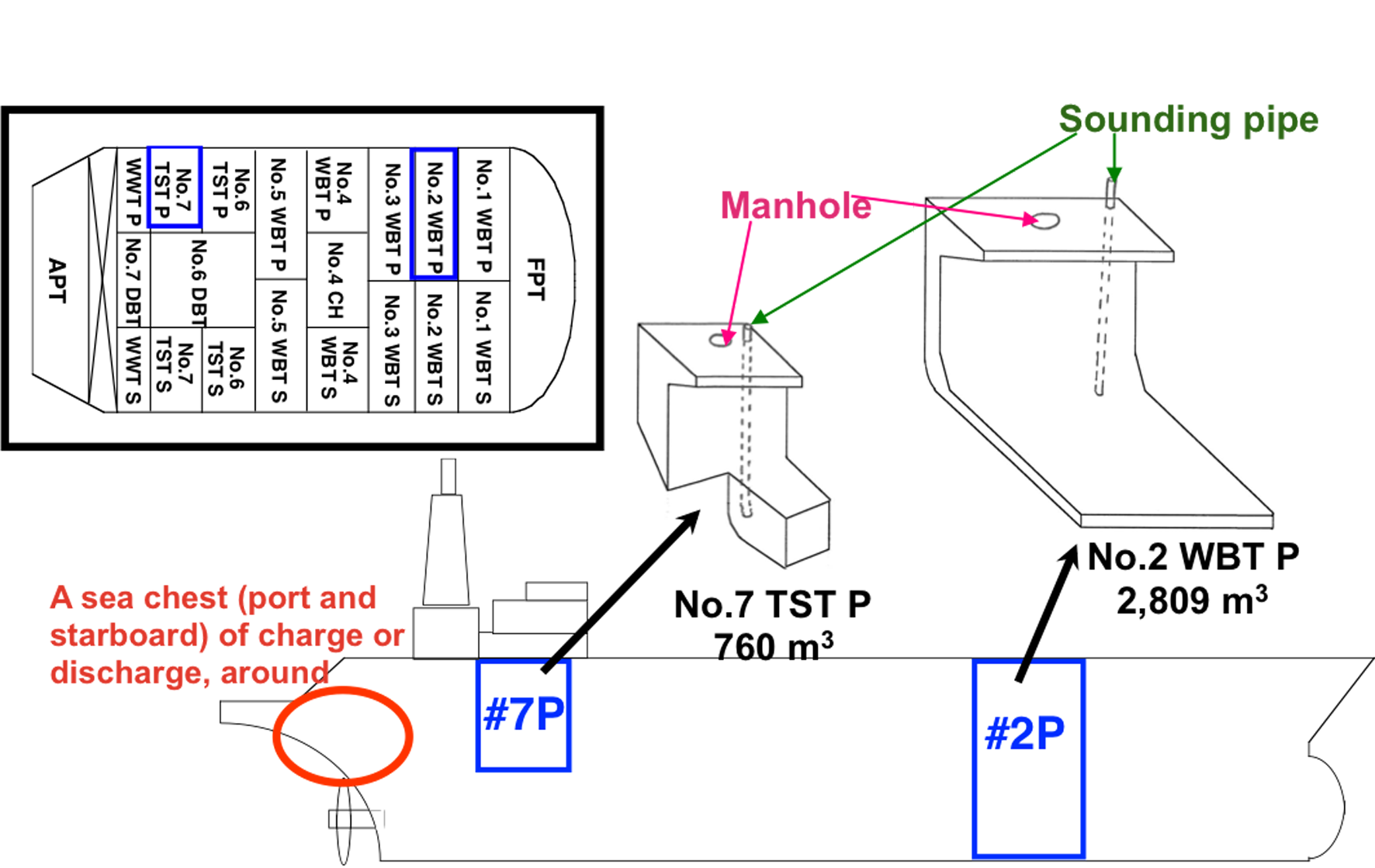

Supplement: Figure S2 — Schematic of ballast water tank sampling. Top side tank (TST), Water ballast tank (WBT), Port (P). (TIF) [file pone.0096274.s002.tif]
